# Editorial: Breast Cancer in Young Women: Dedicated Research Efforts Are Needed

**DOI:** 10.3389/fonc.2022.913167

**Published:** 2022-06-01

**Authors:** Matteo Lambertini, Hee Jeong Kim, Philip Poorvu

**Affiliations:** ^1^ Department of Internal Medicine and Medical Specialties (DiMI), School of Medicine, University of Genova, Genova, Italy; ^2^ Department of Medical Oncology, U.O. Clinica di Oncologia Medica, IRCCS Ospedale Policlinico San Martino, Genova, Italy; ^3^ Department of Surgery, College of Medicine, Asan Medical Center, Seoul, South Korea; ^4^ Department of Medical Oncology, Dana-Farber Cancer Institute, Harvard Medical School, Boston MA, United States

**Keywords:** breast cancer, young women, BRCA, fertility, pregnancy

## Manuscript

Caring for women with newly diagnosed breast cancer at a young age, defined according to international guidelines as ≤ 40 years, is particularly challenging due to its associated additional age-related issues (1). Breast cancer in young women represents approximately one third of the total cases of malignancies in women aged less than 40 years, being the most frequent malignancy and cause of cancer-related death in this group of patients (2) (Silva et al.). The negative prognostic implication of young age at diagnosis may be partially explained by both the lack of screening programs and the higher risk of unfavorable biological features as compared to breast cancer cases in older patients (3). While young age has long been considered a negative prognostic factor, recent data have shown that this seems to be restricted only to patients with hormone receptor-positive disease (4) (Cai et al.). The biological and clinical reasons behind these findings have not been elucidated yet, although suboptimal adjuvant endocrine treatment and lower therapeutic adherence may be considered among the potential explanations (5) (Lu et al.).

Breast cancer in young women is considered a public health problem considering its substantial morbidity and mortality as well as the burden of disparities existing in the care of these patients (6).

While a breast cancer diagnosis at any age can substantially impact on familial relationships and other domains, young women are at a life stage in which additional implications including career, employment and family issues are particularly important. Hence, the potential financial, psychosocial, and social impacts of a breast cancer diagnosis at a young age can be even more burdensome. Importantly, when managing young women with newly diagnosed breast cancer, specific age-related issues should be considered. Among them, genetic counseling, fertility preservation, management of long-term side effects, impact on social and couple relationships and employment are highly relevant. Therefore, the care of young women with breast cancer is particularly complex and a multidisciplinary approach is mandatory (1).

Young age at diagnosis is considered a criteria to refer patients for genetic testing (1). Among different breast cancer susceptibility genes (Wang et al.) and in addition to the implications in terms of screening and risk-reducing strategies (Shraga et al.), identifying a pathogenic variant in *BRCA1* or *BRCA2* genes has clear therapeutic implications in both the early and advanced settings (1). Therefore, more attention is needed to better understand the behavior and outcomes of breast cancer in young women carrying germline *BRCA* pathogenic variants (7). Moreover, the clinical implications of carrying this genetic defect beyond cancer risk require a special focus. Among them, the impact of germline *BRCA* pathogenic variants on women’s ovarian function and fertility are acquiring importance considering their potential negative impact of these defects on the ovarian reserve (8). Considering the current and upcoming availability of new anticancer therapies for the care of these patients, these issues need to be urgently addressed.

Over the past years, increasing attention has been paid to the oncofertility care of young adult cancer patients. As advocated by all guidelines, proper counseling on the risk of anticancer treatment-induced gonadotoxicity is mandatory at diagnosis with all patients with any malignancy and stage diagnosed at reproductive age (9, 10). Being a hormonally-driven form of tumor, there were historically several concerns on the safety and feasibility of managing fertility and pregnancy-related issues in the specific cohort of breast cancer patients. Recent data have contributed to dispel these concerns supporting the safety of accessing fertility preservation strategies prior to starting (neo)adjuvant chemotherapy (11) (Rothé et al.) and of having a pregnancy in women with prior history of breast cancer (12). Nevertheless, some special considerations are needed to manage oncofertility care in young women with breast cancer. Specifically, there are barriers for proper onco-fertility counseling including patients’ side of decision conflict, oncologists’ preference of referral to fertility specialists and standardized protocols of fertility preservation for women with breast cancer, including the preference for adding letrozole a part of controlled ovarian stimulation in order to reduce the rise in estradiol levels during the procedure (9, 10) (Bonardi et al.). The implementation of special oncofertility programs requiring a well organized network between oncology and fertility units are crucial to properly deal with fertility care in young women with breast cancer (Blondeaux et al. and Hours et al.).

The possible diagnosis of breast cancer during pregnancy is another additional possible situation to be considered when caring for young patients (13). This is a challenging condition characterized by several unique medical and psychological needs that require special attention (Costa et al.). Several advances have been made over the past years to better understand the biology of breast cancer arising during pregnancy (Korakiti et al. and Allouch et al.) as well as on the clinical management of this difficult situation (14, 15). Considering the current trend in delaying childbearing, a growing attention is needed to the possible occurrence of breast cancer during pregnancy.

In addition to the potential impact of anticancer therapies on fertility and chances of a subsequent pregnancy, other additional survivorship issues should be considered when caring for young women with breast cancer. Among them, the side effects of endocrine therapy (particularly for the need to administer ovarian function suppression in most of these patients) can be particularly impactful and require dedicated pharmacological and non-pharmacological approaches to counteract them (16) (Choi et al.). Indeed, survivorship is becoming an area of crucial importance in the care of patients with cancer and *ad hoc* programs should be implemented for improving the quality of life of young survivors (17).

With a special series focused on breast cancer in young women (https://www.frontiersin.org/research-topics/13438/breast-cancer-in-young-women), *Frontiers in Oncology* aims at providing updates and news in this field with topics spanning from epidemiology to treatment and its long-term consequences in order to contribute in improving the care of these patients. Further dedicated research efforts are needed to support young women with breast cancer.

## Author Contributions

All authors listed have made a substantial, direct, and intellectual contribution to the work and approved it for publication.

## Funding

ML acknowledges support from the Italian Association for Cancer Research (“Associazione Italiana per la Ricerca sul Cancro”, AIRC; MFAG 2020 ID 24698) and the Italian Ministry of Health (5 x 1000 funds 2017) for supporting his research in the field of breast cancer in young women.

## Conflict of Interest

ML reports advisory role for Roche, Lilly, Novartis, Pfizer, Astrazeneca, MSD, Seagen, Exact Sciences, Gilead and speaker honoraria from Roche, Lilly, Novartis, Pfizer, Sandoz, Ipsen, Libbs, Knight, Takeda outside the submitted work.

The remaining authors declare that the research was conducted in the absence of any commercial or financial relationships that could be construed as a potential conflict of interest.

## Publisher’s Note

All claims expressed in this article are solely those of the authors and do not necessarily represent those of their affiliated organizations, or those of the publisher, the editors and the reviewers. Any product that may be evaluated in this article, or claim that may be made by its manufacturer, is not guaranteed or endorsed by the publisher.

## References

[1] Paluch-Shimon S Cardoso F Partridge AH Abulkhair O Azim HA Bianchi-Micheli G. ESO-ESMO 4th International Consensus Guidelines for Breast Cancer in Young Women (Bcy4). Ann Oncol (2020) 31(6):674–96. doi: 10.1016/j.annonc.2020.03.284 32199930

[2] Fidler MM Gupta S Soerjomataram I Ferlay J Steliarova-Foucher E Bray F. Cancer Incidence and Mortality Among Young Adults Aged 20-39 Years Worldwide in 2012: A Population-Based Study. Lancet Oncol (2017) 18(12):1579–89. doi: 10.1016/S1470-2045(17)30677-0 29111259

[3] Villarreal-Garza C Ferrigno AS de la Garza-Ramos C Barragan-Carrillo R Lambertini M Azim HA. Clinical Utility of Genomic Signatures in Young Breast Cancer Patients: A Systematic Review. NPJ Breast Cancer (2020) 6:46. doi: 10.1038/s41523-020-00188-3 33062888 PMC7519162

[4] Kim HJ Kim S Freedman RA Partridge AH. The Impact of Young Age at Diagnosis (Age <40 Years) on Prognosis Varies by Breast Cancer Subtype: A U.S. SEER Database Analysis. Breast (2022) 61:77–83. doi: 10.1016/j.breast.2021.12.006 34923225 PMC8693310

[5] Lambertini M Blondeaux E Perrone F Del Mastro L. Improving Adjuvant Endocrine Treatment Tailoring in Premenopausal Women With Hormone Receptor-Positive Breast Cancer. J Clin Oncol (2020) 38(12):1258–67. doi: 10.1200/JCO.19.02242 31618128

[6] Rosenberg SM Newman LA Partridge AH. Breast Cancer in Young Women: Rare Disease or Public Health Problem? JAMA Oncol (2015) 1(7):877–8. doi: 10.1001/jamaoncol.2015.2112 26204453

[7] Lambertini M Ceppi M Hamy A-S Caron O Poorvu PD Carrasco E. Clinical Behavior and Outcomes of Breast Cancer in Young Women With Germline BRCA Pathogenic Variants. NPJ Breast Cancer (2021) 7(1):16. doi: 10.1038/s41523-021-00224-w 33579978 PMC7880991

[8] Turan V Lambertini M Lee D-Y Wang E Clatot F Karlan BY. Association of Germline BRCA Pathogenic Variants With Diminished Ovarian Reserve: A Meta-Analysis of Individual Patient-Level Data. J Clin Oncol (2021) 39(18):2016–24. doi: 10.1200/JCO.20.02880 PMC8260903 33891474

[9] Lambertini M Peccatori FA Demeestere I Amant F Wyns C Stukenborg J-B. Fertility Preservation and Post-Treatment Pregnancies in Post-Pubertal Cancer Patients: ESMO Clinical Practice Guidelines†. Ann Oncol (2020) 31(12):1664–78. doi: 10.1016/j.annonc.2020.09.006 32976936

[10] ESHRE Guideline Group on Female Fertility Preservation Anderson RA Amant F Braat D D’Angelo A Chuva de Sousa Lopes SM. ESHRE Guideline: Female Fertility Preservation. Hum Reprod Open (2020) 2020(4):hoaa052. doi: 10.1093/hropen/hoaa052 33225079 PMC7666361

[11] Arecco L Blondeaux E Bruzzone M Ceppi M Latocca MM Marrocco C. Safety of Fertility Preservation Techniques Before and After Anticancer Treatments in Young Women With Breast Cancer: A Systematic Review and Meta-Analysis. Hum Reprod (2022) 37(5):954–68. doi: 10.1093/humrep/deac035 PMC9071231 35220429

[12] Lambertini M Blondeaux E Bruzzone M Perachino M Anderson RA de Azambuja E. Pregnancy After Breast Cancer: A Systematic Review and Meta-Analysis. J Clin Oncol (2021) 39(29):3293–305. doi: 10.1200/JCO.21.00535 34197218

[13] Amant F Lefrère H Borges VF Cardonick E Lambertini M Loibl S. The Definition of Pregnancy-Associated Breast Cancer is Outdated and Should No Longer be Used. Lancet Oncol (2021) 22(6):753–4. doi: 10.1016/S1470-2045(21)00183-2 PMC8868503 34087122

[14] Loibl S Schmidt A Gentilini O Kaufman B Kuhl C Denkert C. Breast Cancer Diagnosed During Pregnancy: Adapting Recent Advances in Breast Cancer Care for Pregnant Patients. JAMA Oncol (2015) 1(8):1145–53. doi: 10.1001/jamaoncol.2015.2413 26247818

[15] Poggio F Tagliamento M Pirrone C Soldato D Conte B Molinelli C. Update on the Management of Breast Cancer During Pregnancy. Cancers (Basel) (2020) 12(12):E3616. doi: 10.3390/cancers12123616 33287242 PMC7761659

[16] Franzoi MA Agostinetto E Perachino M Del Mastro L de Azambuja E Vaz-Luis I. Evidence-Based Approaches for the Management of Side-Effects of Adjuvant Endocrine Therapy in Patients With Breast Cancer. Lancet Oncol (2021) 22(7):e303–13. doi: 10.1016/S1470-2045(20)30666-5 33891888

[17] Partridge AH Ruddy KJ Barry WT Greaney ML Ligibel JA Sprunck-Harrild KM. A Randomized Study to Improve Care for Young Women With Breast Cancer at Community and Academic Medical Oncology Practices in the United States: The Young and Strong Study. Cancer (2019) 125(11):1799–806. doi: 10.1002/cncr.31984 PMC6508998 30707756

